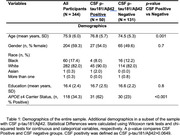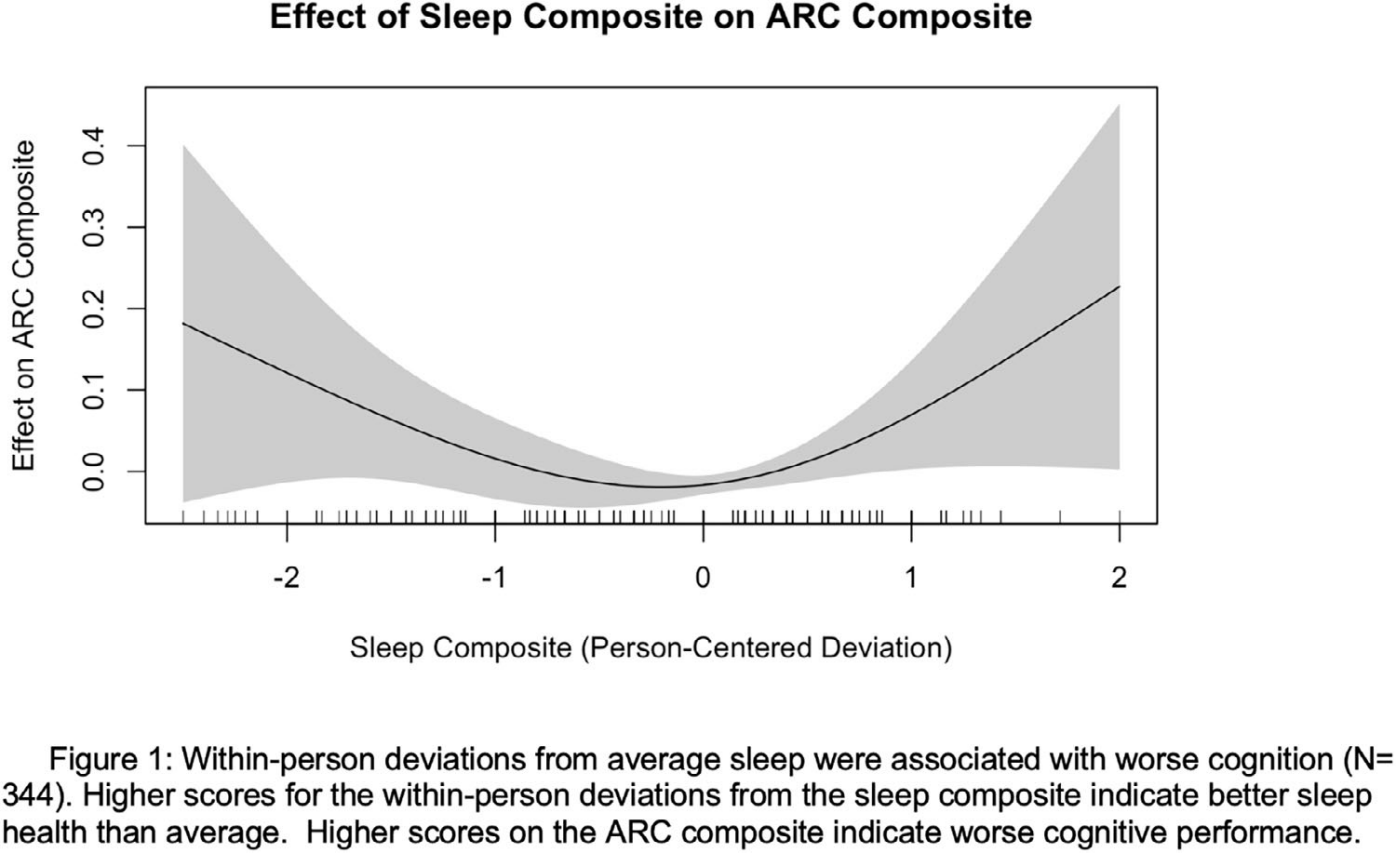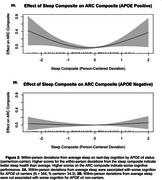# High‐Frequency Smartphone‐Based Assessments Uncover Daily Sleep‐Cognition Associations in Older Adults at Risk for AD

**DOI:** 10.1002/alz70857_105193

**Published:** 2025-12-24

**Authors:** Hannah M Wilks, Matthew S. Welhaf, Andrew J. Aschenbrenner, Brain Carpenter, Brian A. Gordon, Suzanne E. Schindler, Brendan P Lucey, John C. Morris, Jason J. Hassenstab

**Affiliations:** ^1^ Washington University in St. Louis, St. Louis, MO, USA; ^2^ Knight Alzheimer Disease Research Center, St. Louis, MO, USA; ^3^ Knight Alzheimer's Disease Research Center, Washington University in St. Louis, St. Louis, MO, USA; ^4^ Knight Alzheimer Disease Research Center, Washington University School of Medicine, St. Louis, MO, USA; ^5^ Washington University School of Medicine, Saint Louis, MO, USA

## Abstract

**Background:**

Recent advancements in technology have enabled more naturalistic assessments of cognition and sleep in the daily lives of research participants. Questions remain regarding the effects of nightly sleep on next‐day cognition in older adults at risk for Alzheimer's disease (AD). The present study used a novel smartphone‐based approach to examine associations between cognition, sleep, abnormal AD biomarker levels, and genetic risk for AD in cognitively normal older adults.

**Method:**

Cognitively unimpaired participants (*N* = 344, Table 1) were recruited from an ongoing study of aging. Associate memory, processing speed, and spatial working memory were measured daily for several days using the Ambulatory Research in Cognition (ARC) smartphone application. Scores were averaged to create a daily and weekly cognitive composite. Sleep satisfaction, alertness, timing, efficiency, and duration were assessed by self‐report each morning. Cut‐offs were identified for each sleep parameter to characterize good (1) versus poor (0) sleep. Scores were summed to create a daily sleep health composite and averaged to create a weekly sleep health composite. The difference between the daily and weekly sleep health composite characterized nightly deviations from typical sleep.

Linear and generalized additive models determined the relationships between sleep and cognition. Covariates included age, self‐reported gender (male/female), and years of education. *APOE* ε4 status (carrier/non‐carrier), CSF *p*‐tau181/Aβ42 positivity (positive/negative), and day of testing were included, where appropriate.

**Result:**

Within‐person nightly deviations from typical sleep were associated with worse next‐day cognition (*t* (2.7) = 3.5, *p* =  0.035, Figure 1). Deviations from typical sleep were associated with worse next‐day cognition for *APOE* ε4 carriers (t (2.7) = 3.5, *p* =  0.017, Figure 2a); deviations from typical sleep were not associated with next‐day cognition in *APOE* ε4 non‐carriers. Weekly sleep was not associated with weekly cognition. Preclinical AD biomarker status did not alter the relationship between sleep and cognition at the daily and weekly levels.

**Conclusion:**

High‐frequency, multi‐day assessments of cognition and sleep revealed subtle effects of nightly sleep on next‐day cognition in cognitively normal older adults, particularly for *APOE* ε4 carriers. These findings highlight the importance of a multidimensional approach for sleep and cognitive assessments in older adults at risk for AD.